# A Typology of Secondary Stressors Among Refugees of Conflict in the Middle East: The Case of Syrian Refugees in Jordan

**DOI:** 10.1371/currents.dis.4bd3e6437bff47b33ddb9f73cb72f3d8

**Published:** 2018-05-10

**Authors:** Khalifah Alfadhli, John Drury

**Affiliations:** Department of Psychology, King Saud University, Riyadh Saudia, Arabia; School of Psychology, University of Sussex, Brighton, United Kingdom

## Abstract

**Introduction:**

As the years of displacement accumulate, the burden of secondary stressors (i.e., stressors not directly related to war) increase on the shoulders of millions of refugees, who do not have the option of either returning home due to war or having a sustainable livelihood in the host countries. This paper aims to shed light on the overlooked importance of secondary stressors among refugees of conflict in developing countries; it will do this by highlighting the experience of Syrian refugees in Jordan, and developing a typology of these stressors.

**Methods:**

We approached this issue using two levels of exploration. In study 1, we used participant observation and 15 in-depth interviews in Irbid, Jordan. Data were analysed qualitatively using thematic analysis to explore the different types of stressors. In study 2, a questionnaire survey among Syrian refugees in Jordan (n = 305) was used to collect data about a wide range of stressors. Responses were subjected to factor analysis to examine the extent to which the stressors could be organized into different factors.

**Results:**

The thematic analysis suggested three different types of secondary stressors: financial (money related), environmental (exile structures and feelings created by it), and social (directly related to social relations). The factor analysis of the survey data produced a similar typology, where secondary stressors were found to be grouped into four main factors (financial, services, safety, and relations with out-groups). The final result is a typology of 33 secondary stressors organised in three main themes.

**Discussion:**

Syrian refugees in Jordan suffer the most from financial stressors, due to loss of income and high living expenses. Environmental stressors arise from exile and are either circumstantial (e.g., services and legal requirements) or created by this environment (e.g., instability and lack of familiarity). Social stressors were observed among a considerable section of refugees, varying from stressors due to being targeted as a refugee by the locals (e.g., discrimination) to more traumatic stressors that came from both locals and other refugees (e.g., assault). The typology of secondary stressors suggested by the present analysis needs to be investigated in a larger sample of refugees of conflict in other countries in the Middle East, in order to determine its generality. We suggest that it is a basis for a framework for practitioners and academics working with refugees in the region.

**Key words:**

Armed conflict, Syrian, Jordan, refugees, forced displacement, exile, daily stressors, secondary stressors, trauma, typology.

## Introduction

The aim of this paper is to identify the stressors arising from the exile environment among refugees of conflict in the Middle East and to explore the relationship between different stressors, in order to provide the foundation for classification and measurement of such stressors that could be helpful to both researchers and practitioners working with refugees in the region. To achieve this goal, we first conducted a qualitative study (ethnography) that included observations and 15 in-depth interviews with Syrian refugees and local relief workers in Jordan to identify the main themes of such stressors. The second study was a survey (n = 305) among the same population to get a sense of the most prominent stressors and to validate the thematic structure of study 1, in addition to identifying factors of related stressors that can be used as a first step towards building a scale for assessing secondary stressors in the region. Before describing the research we provide the background and explain the need for our analysis.

**The current refugee crises**

The United Nations High Commissioner for Refugees (UNHCR) global trends report for 2015^1^ shows that the current forced displacement crisis is the largest in modern history, exceeding the situation at the end of the Second World War. More than 65 million people have been forced to leave their homes, and 86% of them are hosted in developing countries^1^. These people are not expected to return home soon, as the current forced displacement is characterised by a prolonged duration, where refugees spend an average of 26 years in host countries^1^.

Jordan is an example of the refugees’ impact on countries in areas of armed conflict like the Middle East. Jordan’s current population is 7.5 million, including 656,170 Syrian refugees who have arrived since 2012^2^, and 2,117,361 Palestinian^3^ refugees who were forced to leave their country by the Israeli occupation, more than 50 years ago^4^. However, as with many countries in the region, Jordan has not signed the 1951 Refugee Convention^5^.

The international response to the forced displacement crisis falls short in meeting the persistent challenges facing the majority of refugees, where funding for the relief responses in developing countries is far from meeting the needs in the areas where demand is high. For example, the humanitarian funding appeal in Jordan in 2016 covered only 22% of projected costs.^6^ This shortfall leaves refugees with little support in facing stressors associated with prolonged displacement, which increases the negative impact of such stressors on their well-being.

**War trauma and distress, in context**

When thinking about refugees of war, it is easy to recall many mainstream media images of the trauma of war forcing refugees to flee their homes. However, the untold story of exile is different as “affected people often identify as their greatest sources of distress, not the memories of past violence and horrific events but the problems of everyday living” (7 p. 8). The World Health Organisation and International Medical Corps^8^ undertook an assessment of the mental health and psychosocial needs of Syrian refugees (n = 7964) in different regions of Jordan. They found that the most common mental health concern reported by the refugees was “Worry and concern over the situation, relatives and the future”, followed by “Fear from environmental threats”. When asked about the help they needed, the top priority was “To improve services and living conditions”, while only 13% expressed a need for “Counselling or psychological support”.

Recent studies have found a relatively high prevalence of post-traumatic stress disorder (PTSD) among Syrian refugees in neighbouring countries.^9^ However, for a more contextualised understanding of the status of trauma among Syrian refugees, we should take into consideration that there are factors other than war exposure that contributes to PTSD. Rather, some of these arise from the exile environment. Al-Smadi et al.’s study^10^ shows low scores for PTSD among the majority of Syrian refugees in Jordan, and that the higher scores are associated with factors not directly related to war - chronic disease diagnosed in exile. Basheti, Qunaibi, and Malas^11^ also found an association between psychological distress and living conditions; refugees in tents had double the rates of refugees in caravans. The previous literature is best summarised by Miller and Rasmussen^12^ who argued that “daily stressors” have a direct effect on mental health among refugees of conflict, in addition to increasing the negative effect of trauma on mental health. This conclusion also supported by later studies (e.g., 13^,^^14^^,^^15^**).**

**Examples of stressors**

Stressors arising from the exile environment cover a wide range of challenges among refugees from different regions, although some of these stressors are sensitive to context. The literature on refugees of conflicts in developing countries provides us with examples of such stressors: among refugees in Afghanistan, these include overcrowded housing, poverty, unemployment, the security situation, violence in the home, poor health and pollution^16^; El-Shaarawi^17^ reports inaccessibility to (work, education, and health) services, social isolation and separation from family and friends, decline in living standards, in addition to a status of uncertainty about the future among Iraqi refugees in Egypt. Even using unfamiliar transportation was found to be stressful, as in the case of Darfur refugees in Sudan.^18^

**Secondary or daily stressors**

Some studies among refugees of conflict use the term “daily stressors” in referring to stressors arising from the exile environment (e.g., 12^,^^16^), which we suggest is problematic for two reasons. First, some of these stressors are not daily (e.g., physical attack). Second, the term is sometimes used in the literature in contrast with traumatic stressors although some of the “daily stressors” can be traumatic (e.g., domestic violence). Therefore, we recommend using the term “secondary stressors”, as found in the disasters literature, which describes this group of stressors as “continuing or chronic problems that ... are not directly related to or inherent in the event” (19^,^ p. 3). For this study, we define secondary stressors as stressors not directly related to war (arising from exile environment), and which are socially mediated in nature (operating through the societal response - or lack of societal response - to the events).

**Goals of the paper**

Based on the literature showing that secondary stressors among refugees of conflict are important but receiving less attention, and the fact that the few examples of research that focus on them were conducted outside the Middle East when the Syrian crises is a main contributor of the current global displacement crisis, we decided to focus on Syrian refugees in Jordan (which is a major refugee host country in that region) to explore the forms of secondary stressors among them, in two stages. In study 1, using an ethnographic framework we conducted in-depth interviews to identify types of secondary stressors and common themes between them. In study 2, we undertook a survey (n = 305) in order to examine the relation between stressors (factor analysis), in addition to assessing the most common stressors among the refugees.

In addition to advocating using the term “secondary stressors” to describe the stressors facing refugees of conflict in exile, the typology we developed can be helpful to both practitioners and academics working with the refugees of conflict in developing countries, by identifying the specific domains that the refugees are in need of support, and the relation between stressors.

## Study 1

The aim of study 1 was to identify some of the range of stressors that the Syrian refugees in Jordan encounter, some of which could be specific and sensitive to the setting. To achieve that, we conducted an 8-month ethnography among a community of Syrian refugees, where we collected contextual data from trust-based in-depth interviews and participant observation by the first author.

## Method

**Participants.** The interviews (n = 15) all took place in the northern region of Jordan, by the Syrian borders. Most of the participants were from the nearby Syrian region of Dara’a, which is on the southern Syrian borders with Jordan, and were recruited through the first author who served as a volunteer in a neighbourhood heavily occupied by Syrian refugees. We interviewed 13 refugees who were residents of the neighbourhood, in addition to two local relief workers who worked with Syrian refugees for several years. Only three interviews were with women, due to the local culture restricting private interaction between females and males (the first author being male).

**Procedure.** The first author collected data in the form of in-depth interviews and participant observations, during his 8-month period as a volunteer in a school located in a neighbourhood predominantly inhabited by Syrian refugees. The first author made it clear (whenever meeting someone new, in addition to later reminders) to the school management, other volunteers and the community members that the main purpose of being there was to collect data in the forms of observations and interviews.

The interviews were undertaken after establishing trust-based relations and then snowballing recruitment. Interviews lasted between 40 minutes to one hour and were conducted in Arabic and audio recorded using the first author’s phone. The researcher (who is a native Arabic speaker) transcribed the interviews and then translated them to English. The researcher also kept a regular diary (21,062 words) that included information obtained through casual chats and observations he made in the neighbourhood, in Syrian family homes, and while accompanying the refugees through activities such as shopping using food coupons and getting necessary documents.

**Analysis.** Due to the exploratory nature of this study, which aimed to lay out a foundation for a typology of secondary stressors, we adopted a grounded approach (bottom-up) to analyse the data. We used Big Q thematic analysis, which is flexible and involves an organic coding process.^20^ We started coding the interview data from the lowest level possible and then identified relations between codes themes and merged or split some of them where needed. The extracts and observations we present in this study are chosen because they are representative of statements found in the dataset.

**Ethical review.** A written consent was obtained from the interview participants. This study (ER/KHTA20/3) have been approved by the Sciences & Technology Cross-Schools Research Ethics Committee at University of Sussex (crecscitec@sussex.ac.uk).

## Results

We identified three main groups of secondary stressors (financial, environmental and social) among the participants, which they suggested were common among Syrian refugees in Jordan. Consistent with the literature^21^^,^ the interviews suggested that these participants struggled more with the secondary stressors than the memory of war or its direct effect on their life (e.g., deaths of relatives in Syria).

**Financial Stressors**

This group of stressors is a result of the forced displacement of refugees from subsidised Syria to the relatively more expensive Jordan, losing their previous sources of income, and facing challenges finding jobs in exile due to restrictions on refugees’ ability to work. We identified poverty as the main financial stressor that most of the participants faced, in addition to three other stressors (residence, education and health) that arose from the financial strain that the refugees faced.

**Poverty.** Syrian refugees left their homes to travel to Jordan in order to escape from a life-threatening military operation and deteriorating basic services, with the hope that their forced displacement would be for only a few months:

We came [to Jordan] on a temporary base. We couldn’t wait to get back. I even did not say goodbye to my family. We were thinking 2-3 months maximum.

Participant 7

With the priority to save life and as a temporary solution, many of Syrian refugees walked away from their jobs, farms and shops across the borders with Jordan with little savings:

Even if you have some savings it will be gone, especially when you are not allowed to work [pause] The financial ability of people diminished as the crisis prolonged.

Participant 9

In addition, the Syrian refugees faced a serious financial challenge as they moved from less expensive ‘socialist’ Syria (where basic services were subsidised by the government) to more expensive free market Jordan, and had to face additional inflation caused by hundreds of thousands of refugees who moved out from self-owned houses in Syria to rent limited housing units in Jordan for high prices:

We did not have to worry about rent, electricity and water bills. [pause] We had free education and health services [pause] Here everything needs money.

Participant 2

Refugees have little financial support in facing such challenges, as they depend on the UNHCR which provides food coupons (around $21 per person monthly) for registered refugees, in addition to some irregular aid from charities:

In the beginning, the aid from UN was available [pause] it was fine, but now it’s less. Instead of 24JODs($33)/person, now they give 10-15JODs($14-$21) or even cancelled it for some people. I still receive the [food] coupons 15JODs($21), which are only enough to provide us for 20 days. The rest of the month we have to take loans from friends or find any work to survive. Each month, we receive a text message to inform us that the coupon debit card is charged, then we go to the authorized outlets to buy grocery. We are only allowed to buy food [using the coupons].

Participant 2

A smaller group of refugees receive a monthly allowance after being evaluated and approved by home visits undertken by a team from UNHCR, although such payments are not reliable. The refugees call it “the retina” because the payment is through ATMs with a retina scanner:

We receive 100JODs($141) to pay toward the rent, that’s all [pause] the Retina [monthly salary][pause]. They came to evaluate our home, twice in the same month [pause] two persons each time. They evaluated and approved us, but then it was taken away. So we went back struggling with the rent. This is an on-going suffering. The monthly salary was cut off for 4 months and then returned, but they have cut it again now.

Participant 8

During the first author’s visits, he noticed the simple furniture in the crowded homes of Syrian refugees, and torn clothes on some of the children. In addition, there were examples of how living in poverty can increase expenses, like in the case of one Syrian family caught in a situation where they were using most of their income to pay rent for a furnished apartment, which made them unable to save money and buy furniture and move to a less expensive unfurnished apartment (observation made on March 21st, 2016).

**Residence. **Due to the influx of hundreds of thousands of Syrian refugees, there was a scarcity of housing units that led to an increase in rents. Many refugee families had to share housing with each other, especially upon arriving. A refugee described her experience of staying in her husband’s brother’s two-bedroom apartment, when they arrived from Syria:

When we first came to live with my older brother in law, he was living with his wife and kids. So, we lived with other two families in one apartment, which was very hard. It was only two-bedroom apartment with a small living room, one toilet and a small kitchen [pause] The older brother had a wife and two kids, we had three kids and the youngest brother had a pregnant wife. [pause] 11 persons.

Participant 7

Sharing small apartments is a practice that Syrian families sometimes continued to do not just on a temporary basis upon the arrival of a new family, but also as a permanent solution to reduce the cost of rent and utilities. The first author visited many Syrian families’ homes and in one case, there was an extended family of ten people who had been living for years in a three bedroom apartment, including their married son and divorced daughter with their own children (observation made on February 7, 2016). Due to the high rents, refugees move to a cheaper apartment whenever possible (especially with little furniture), even if the place is not well maintained or is affected by pollution (e.g., burning garbage and flooded sewers) (multiple observations).

**Education.** Basic education (up to 12th grade) is available for Syrian refugees in Jordan free of charge, but challenges facing Syrian students in the Jordanian government schools pushed two participants to consider private school as an option:

Education is expensive in Jordan because government schools do not take care of refugee kids. They go to school in the afternoon and get little attention [pause] I pay 45JODs($63) a month for my son’s private schools. It is a burden, but I’m handling it.

Participant 3

Also, there was the stress of sending children to college; this was an aspect that they did not have to worry abut in Syria, where free college was offered to all citizens:

My older son is 19 years old and finished 10th grade in Syria, but now he cannot go to school because he’s working full time. He didn’t finish high school because I don’t have the money to send him to college.

Participant 2

**Health.** Nine interviewees said that they were satisfied with the health services available to them, especially after they registered with UNHCR. However, three indicated that that health services available to them were basic and even the medical missions and subsidised private health services could not cover some of their serious health needs:

I have serious health issues that the [public] health service did not treat. I need serious medical care, which is expensive.

Participant 6

One participant complained about discrimination in providing health care for Syrian refugees in specific health centres, which he believed was sub-standard:

When I get sick I don’t go to the doctor. I have no medical insurance. There is a health centre in [location] for refugees. Once I took my friend there and it was very bad. They treated us like cockroaches, not patients [pause] openly, everybody there did. Even if I was dying, I won’t go there. Sometimes I have health problems and go to a docto, who is the mother of a Jordanian friend.

Participant 4

**Environmental Stressors**

This group of stressors have in common that they arise either from the circumstances of the exile environment (official documentation issues) or a general feeling created by this new environment (instability and lack of familiarity).

**Official stressors.** Some of the Syrian refugees arrived in Jordan through the official borders gates with Syria (closed since April 2015^22^^,^^23^**)**, while others arrived through the border fence and then on to refugee camps. Refugees had the option of leaving the camp if they secured a Jordanian guarantor. However, some refugees had their own ways of leaving the camp, as reported by one of the participants:

My family entered Jordan using passports, but that was dangerous for me. So, I entered through the border fence with the help of Free Syrian Army. Jordanians [Jordanian Army] took us to Alzaatri refugee camp, where I left in the same day with smugglers for 70JODs($98). Three months later I came back to the camp with a guarantor [to issue official documents]. I did not know him [the guarantor], there were people would arrange it for 50JODs($70)

Participant 10

UNHCR registration was important for many refugees because it was a requirement to receive aid and services provided by the government or the NGOs. At the peak of the influx of refugees, the wait for a UNHCR registration interview appointment was months:

The situation went from bad to worse. We were not receiving food coupons, because we had to wait for a UNHCR [registration]appointment due to huge numbers [of refugees]. It was easier for people who came earlier. We arrived in August but had to wait until December to register with UNHCR.

Participant 7

It is worth mentioning that UNHCR registration should be renewed annually:

The UNHCR registration has to be renewed every year, while the Jordanian ID [for refugees] does not expire.

Participant 2

Many services require a valid UNHCR registration and one example is a health centre that had signs explaining that blood tests will not be done without a valid (up-to-date) UNHCR registration (observation made on 9th of February 2016).

The UNHCR subsidizes the cost of refugees’ health care, which could be the reason for asking for a valid UNHCR registration as a requirement to receive health care services:

… let’s take health services as an example, Syrian [health care expenses] are paid by the UNHCR while the Jordanian [expenses] are paid by the ministry of health

Participant 15 (a local relief worker)

However, being registered with the UNHCR is not the end of the struggle with documentation issues, as the refugees are required to acquire other legal documents. This realisation came to the first author after a short period in Jordan when he was walking with a Syrian student when he asked the first author to avoid passing by a policeman. The first author was puzzled, as he knew that all students in the school were registered with UNHCR, but the other children told him that the student did not have “the ID” (observation made on December 16, 2015). A few months later, the boy’s family was deported.

The Jordanian government issued a “security identity card” for Syrian refugees and required them to carry it and show it at official departments and at security checkpoints. The Jordanian security identity card has been updated with a new magnetic one, which contains the refugee’s retina scan information. A refugee explains the process and the requirements:

They [Jordanian Government] required us to have the white identity card at first. I gave them my [Syrian] family card and they granted me the white card. Then they asked us to get the magnetic identity card, which requires having the white card. It was a matter of time due to pressure. We also had to do a blood test, which I paid 30JODs($42) for, in the [name] hospital last August. I’ve heard that the price was reduced to 5JODs($7). Only adults have to do the test. I took the result to the nearest police station and got the new magnetic security card. They also asked for UNHCR registration and a certified rent lease contract.

Participant 7

Some refugees did not get the new identity card because of the high cost of the blood test, which used to cost 30JOD($42) per person before it was subsidised to 5JODs($7). However, not holding the new Jordanian identity card can lead to serious consequences, as a participant explains:

In a few months, they would not register my kids in the school, for the next year. You need it to goto health centres and hospitals. If I wanted to travel from Irbid to Amman [the capital] I need it, otherwise I would get into trouble [pause] I would be legally questioned by the police patrol [road block] [...] They might just give you a warning, depend on the police officer [pause] they might throw you in al Azraq camp [more of a detention camp] or let you go. Especially if you talked back, they could throw you back to Syria.

Participant 2

Syrian refugees in Jordan are only allowed to work if they have a working permit.^24^ They find it hard to get a permit, and face serious consequences if they have worked without one:

They [refugees] don’t have working permit. They cannot have a permit. Anytime they could be caught and deported. Syrian refugees don’t get a permit. We tried and it didn’t work.

Participant 5

A local relief worker noticed an additional reason for Syrians not to get a working permit:

The Syrian refugees try to stay away from getting a working permit because it restricts them to do just one specific job (e.g. builder or sweets maker), while the refugees try to work in more than one job to compensate for the low wage. One day he works in a farm, next day he will work as a builder.

Participant 15

However, Syrian refugees have ways around restrictions on work, as the first author noticed that some refugees chose to work night shifts, outside the working hours of Ministry of Labour inspectors. Other refugees decided to send their children to work (they would not get deported if caught) for shops and restaurants for very low pay (less than $3/day) (observation made on May 15th, 2016 ).

**New Environment**

Many of the Syrian refugees in Jordan come from the southern region and settle in the northern region of Jordan, due to the proximity (20 kilometres) and similarities in culture. However, Syrian refugees in many cases found themselves in a new environment where they felt disoriented, especially those who came from rural areas and settled in a city:

We did not know the laws, refugee regulations. If I had a sick child, I did not where to go. I have diabetes and high blood pressure and did not know where to get medicine.

Participant 10

This new environment creates a lack of familiarity that could have a deep impact on a newly arrived refugee, who feels a deep sense of estrangement and powerlessness:

I was walking back home when I got lost. It was hard to go from one area to another, without knowing where you should go! You take a taxi that asks for 5JODs($7), but you don’t know if that’s too much or not. You don’t know the value of the 5JODs, or how long the distance is!

Participant 12

**Instability.** The participants expressed a deep feeling of instability and loss of aspiration of the future. This is mainly because they consider their stay in Jordan as being temporary, especially when they do not have the option of permanent residence. They felt deep instability that pushed some of them to consider relocating to a third country or even returning to Syria:

The biggest struggle for a refugee is the temporary situation. Where are we going? then what? the instability!

Participant 7

Another effect of the exile environment on Syrian refugees was the lost sense of normal life, and one clear example of this lost normality can be found at bedtime. As most refugees are not allowed to work and their children go to schools after the Jordanian students finish school, they seem to adopt a nocturnal lifestyle where the whole family stays up late (multiple observations).

**Social Stressors**

Although many of the environmental and financial stressors are socially mediated, we labelled this last group of stressors as ‘social stressors’, because social relations or forms of social interaction (or lack of interaction) are the main source of these stresses.

**Separation from relatives. **The participants belonged to a collectivist culture, so it was not a surprise that they showed great concern for their relatives beyond the borders, in Syria and other countries:

There was suffering [pause] to stay away from my family. [pause] I have a brother in Syria and another brother in Libya. Like the destiny of the Syrian family, we are scattered all over the world.

Participant 3

**Relations with host-community**

Across ten of the interviews, refugees mentioned the word “Jordanians” nine times in a positive context, nine times in a negative context and three times in a neutral context, which may indicate that the relation between Syrian refugees and their Jordanian hosts is a love-hate relationship, were refugees have a mix of negative and positive experiences with locals and local authorities (e.g., Participant 4, who was treated badly at the health centre, but have very supportive Jordanian employer). The general perception of the relationship seemed fine, but there were still everyday indications that Syrians were no longer welcome. One refugee attributed it to the commercial aspect of the crisis:

At first, people were very welcoming to Syrians and treated them very well, but with the increase of influx, things turned to be materialistic. At the beginning, Syrian families were offered housing for free, then turned to rent only. My first landlord even used to bring me breakfast and pick some fruits for me.

Participant 9

On level of personal relations, the picture is more positive in that we found reference to many strongly supportive relations:

It was struggling until we managed to know good people. Every community has good and bad people [pause] My Jordanian co-workers are a good example. They visit me and I visit them, which made me no longer feel as a stranger. I have excellent relationships with them, which reduced the struggles I have.

Participant 3

the owner pays all [working permit] fees [pause] He [Jordanian employer] told me not to worry about it, even if it’s 600JDs ($846) [a year], he would pay it [pause] Even if it goes to a 1000JDs ($1410), he would still pays it. This is hard to find anywhere els.

Participant 4

**Prejudice.** Some participants reported verbal and physical attacks, and while they stressed that these were rare,they felt they were targeted because of their refugee status:

We’ve been treated somewhat bad. Jordanians do not accept Syrians. They don’t treat Syrians well.

Participant 8

However, it seems that some refugees blamed other refugees for such treatment:

They think Syrian women are cheap. Some Syrian women caused this reputation, but it annoys me when they generalize it.

Participant 7

**Discrimination.** Some interviewees said that they felt welcomed by Jordanians, who treated them well on both personal and official levels. However, they also reported some aspects of discrimination:

At the airport, everybody enters with dignity, except Syrians. They treat him like if he has a bomb in his bag.

Participant 1

Some Jordanians apparently held a belief that refugees should have lower status, and they treated them accordingly. The first author witnessed this first-hand while sharing a taxi with a Jordanian, who said during the ride said that Jordan gave a lot to the Syrian refugees who are ungrateful and have a strong voice. He used a traditional proverb “a guest with a sword” to illustrate that the refugees are not at the same level as Jordanians and should behave accordingly (observation made on February 19th, 2016).

**Exploitation.** Some interviewees reported exploitation practices that targeted refugees either by taking advantage of them based on their identity or needs as refugees (e.g., work and housing):

When I want to rent a place I suffer [pause] either they raise the rent or stuff like that. Even a taxi driver when he hears my Syrian dialect asks for more. [pause] Bullying. This does not happens to Jordanians [pause] The salary [for Syrians] is too low compared to other workers, even to the minimum wage set by the government -which was 180JODs($253)/month-. They offered me 150JODs($211) for working 10 hours. The 180 minimum is for 6 hours! Take it or leave it.

Participant 4

There was a restaurant in the neighbourhood where a Syrian worker resigned but did not get paid, and the first author witnessed when a group of Jordanian young men threatening the restaurant’s manager to pay the former worker his unpaid salaries. Once they left, the manager called the restaurant’s owner and told him that he will “get rid of the worker and send him back to Syria" (observation made on May 16th, 2016).

## Discussion

Syrian refugee interviewees reported the issues that they face in exile and that challenge them the most. Occasionally, some refugees reported primary stressors caused by war (like loss of relatives or property back home), but the main challenges were those that they have to face in exile (secondary stressors).

We found three main sources for stressors among the refugees in exile; first, financial stressors where the refugees lost income and found themselves in a relatively expensive host country, which led to poverty and other struggles caused by that poverty (crowded housing, education, and health stressors); second, environmental stressors created by the circumstances of exile (e.g., documentation issues) and the feeling created by this environment (unfamiliarity and instability); third, social stressors where social interactions are the main source of stressors like prejudice, discrimination, and exploitation.

Secondary stressors can be intense and could lead to traumatic experiences, which led some refugees to consider returning to war-ravaged Syria, as in the case of Participant 5 who was struggling for years to provide for his family without any improvement in living conditions, and Participant 14 who was a single mother with three disabled children, with no stable income.

Establishing personal relations with community members, and providing contributions as a teacher and a part-time relief worker helped the first author establish trust for uncensored and safe disclosure during the interviews and casual chats with refugees and volunteers. The immersion of the researcher in the community for a relatively long time allowed him to make sufficient observations to avoid a superficial and restricted perspective of the community that a passing observer could be exposed to. One example of such a superficial and partial image of the community was experienced by the first author once he arrived in the city. He encountered Syrian boys begging on the main street, while the neighbourhood behind that same street (200m away) included Syrian families, who we found out later had to be convinced to accept aid..

However, our approach had some drawbacks in regard to face-to-face communication, which limited interaction with females due to cultural restrictions with the male first author and also made it harder to discuss sensitive issues like sexual harassment. (One female participant mentioned harassment but did not use the word “sexual” directly and a male participant reported that it happened without discussing specific cases.)

## Study 2

In order to advance our exploration of secondary stressors among Syrian refugees in Jordan, we conducted a questionnaire survey of secondary stressors among a convenience sample of Syrian refugees in Jordan. This study first aimed to get a sense of how common the stressors are that we identified in the previous study; its second objective was to test and validate the themes resulting from qualitative analysis; its third intention was to detect any other stressors that could have been missed by the ethnographic case study (e.g., rare, unobservable or not accessible face-to-face).

## Method

**Participants.** The participants in this study were 305 Syrian adults (18 years and above) who left Syria because of the war and were now living in Jordan. The majority of the participants were male (64.3%).

**Building the questionnaire items.** The survey items were created using multiple sources, mainly guided by the qualitative data from study 1, and then consulting local experts (two academics and one relief worker) who worked with Syrian refugees in Jordan. Finally, we drew upon other sources relevant to the refugees’ situation^16^ or others from different context.^19^ The survey included 51 questions (see Table 1), each asking how frequently the respondent was exposed to the secondary stressors (with a 5-point Likert scale, from ‘never’ to ‘always’) for all the items, except for seven items (18,38,40,42,44,46 and 48) which expressed the degree to which they agreed with a statement (on a 5-point Likert scale, from ‘totally agree’ to ‘totally disagree’). At the end, the questionnaire included nine demographic questions. The survey questions were translated to Arabic by the first author (who is a native Arabic speaker) and then back translated and checked against the English questions by a research assistant (who was English-Arabic bilingual).

**Recruitment and data collection. **In September 2016, we used an online survey service (Qualtrics) to construct the survey. We recruited participants using Facebook, based on our knowledge that Syrian refugees are widely using the site to stay connected to their family and friends who were separated due to war, in addition to sharing information about services in exile. Refugees access Facebook using phones, so we designed the online survey to be mobile-friendly. The first author used the social relationships that he built during the ethnography in Jordan with refugees and community organisations to spread the word about the survey, in addition to sending invitations through public Facebook groups dedicated to Syrians in Jordan. We sent link invitations to people with public profiles with the following criteria: adult, Syrian, and living in Jordan.

**Analysis.** We started with basic descriptives of the sample characteristics, followed by the percentages that show how common the different stressors are among the participants. Finally, we conducted a principal factor analysis (with Varimax rotation) to identify possible groups of stressors that are related to each other and independent from other stressors.

**Ethical review.** A page of participant information and a consent was obtained from the online survey participants by answering a clear question confirming their consent to participate. This study (ER/KHTA20/4) have been approved by the Sciences & Technology Cross-Schools Research Ethics Committee at University of Sussex (crecscitec@sussex.ac.uk).

## Results

The sample (n = 305) was fairly young (more than 60% < 30 years old) and almost half of them (47.7%) were single, which could be due to the recruitment method used, whereby Facebook users among the refugee community tend to be younger. Furthermore, it is worth mentioning that the majority of participants (84.8%) spent three years or more in Jordan (due to the closure of the border), which we expected to establish among them a good awareness of secondary stressors of prolonged displacement.

**Figure:**
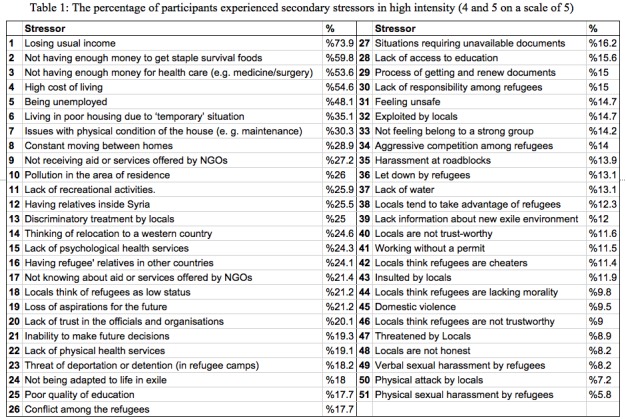

In Table 1, we find a percentage analysis that highlight the most common stressors that sample of Syrian refugees suffered from in high intensity (“most of the time” and “all of the time”). On the top of the list we find the financial stressors that the majority of refugees suffer from (e.g., 73.9% lost usual income), while the structural environmental stressors appears to affect a large percentage (quarter to third) of the participants. The stressors regarding the relation between The Syrian refugees and the locals were reasonably low (<15%).

Although the previous analysis gives, us a snapshot of the most common stressors among the refugees, it might fail to detect the importance of some highly sensitive social and political stressors (e.g., sexual assault and working without a permit), where refugees might be hesitant to report encountering such stressors. Also, this approach cannot detect some serious (high intensity) stressors (e.g., domestic violence) that only occurs to some refugees, but nevertheless should be highlighted in order to be addressed. To highlight such underlying stressors and explore the relations between the general stressors we conducted an exploratory factor analysis (EFA).

**Factor Analysis.** A principal factor analysis was conducted on the secondary stressors items with Varimax rotation. The Kaiser-Meyer-Olkin (KMO) measure was 0.73; all KMO values for individual items were greater than .64, which is above the acceptable limit of .5 which indicates an acceptable sample size.^25^

An initial analysis was run to obtain eigenvalues for each factor in the data; 15 items fall in four factors had eigenvalues greater than 1 and in combination explained 60% of the variance. After rotation, the three factors retained corresponded to four dimensions of secondary stressors. Reliability scores for most factors were acceptable: secondary stressors α = .67; financial stressors α = .86; stressors related to services α = .72; safety stressors α = .72; and relations with locals stressors α = .5

## 

**Figure:**
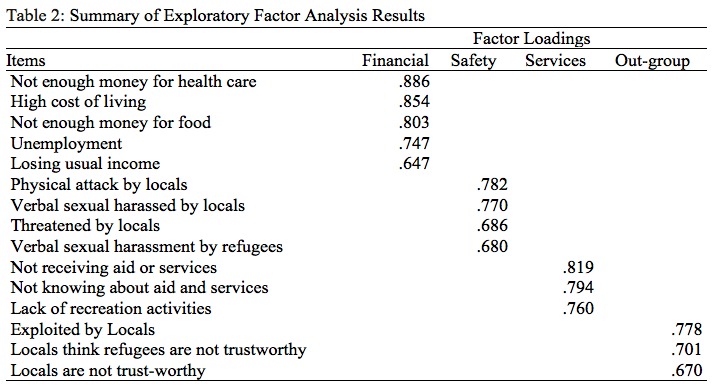

## Discussion

As expected, financial stressors were the most common severe stressors among the participants, due to the lack of financial support and restrictions on employment of refugees and expensive life in Jordan. The factor analysis identified four coherent factors. The low reliability of the factor for relations with the locals (Jordanians) factor could be due to the sensitivity of the questions, and thus the responses of some participants. This suggestion is based on observations by the first author during interviews and casual conversations, where most of the Syrian refugees were very careful not to talk in a negative way about Jordanians, to avoid being seen as criticising their hosts. The relation between the Syrians and Jordanian is relatively good, but during casual talks with refugees who were close to the researcher, some of them reported negative encounters with Jordanians that left an impact on them. It is possible that some of the refugees who had negative experiences with Jordanians did not report it in the questionnaires due to two possible factors: first, because of cultural traditions that consider it highly inappropriate to criticise your host; and second, due to the political status quo where refugees suffer from vulnerability in relation to locals and the government (e.g. stressors 20, 27, 29, 35).

## General Discussion

We found that the qualitative analysis produced three main themes or types of stressors, which were financial, environmental and social, while the quantitative analysis produced four themes or types of stressors, which were financial, services, relation with locals and safety. We identified that the quantitative themes broadly map into the three main types of secondary stressors that resulted from the qualitative analysis. The financial stressors theme was matched in both analyses, services can perhaps be considered as a sub-type of a broader environmental category, and relations with locals and violence can be considered sub-types of the main social stressors category.

Based on the two studies described above, we therefore propose a typology of 33 secondary stressors (Table 3) that is organized into three main themes (financial, environmental and social stressors). The selection of these reflects, first, the qualitative analysis that generated the three main themes; second, the factor analysis that generated four factors, which map onto the three qualitative themes; and third the percentage of each stressors among the sample. The scale of fifteen secondary stressors items (Table 2) is therefore situated within the typology, with its four sub-scales (financial, services, relations with locals ,and safety stressors).

Financial stressors consistently appeared as a top priority in both the qualitative and quantitative analysis, while there were less frequent - but not less serious - issues that seek our attention (e.g. 5.8% reported physical sexual harassment by another refugee). We had indirect reports (not using the word “sexual” and second-hand experiences) of sexual harassment from two participants during the interviews, but, combined with the fact that 17 participants in the survey reported being sexually harassed, we can conclude that these were not isolated incidents among the refugee community.

**Figure:**
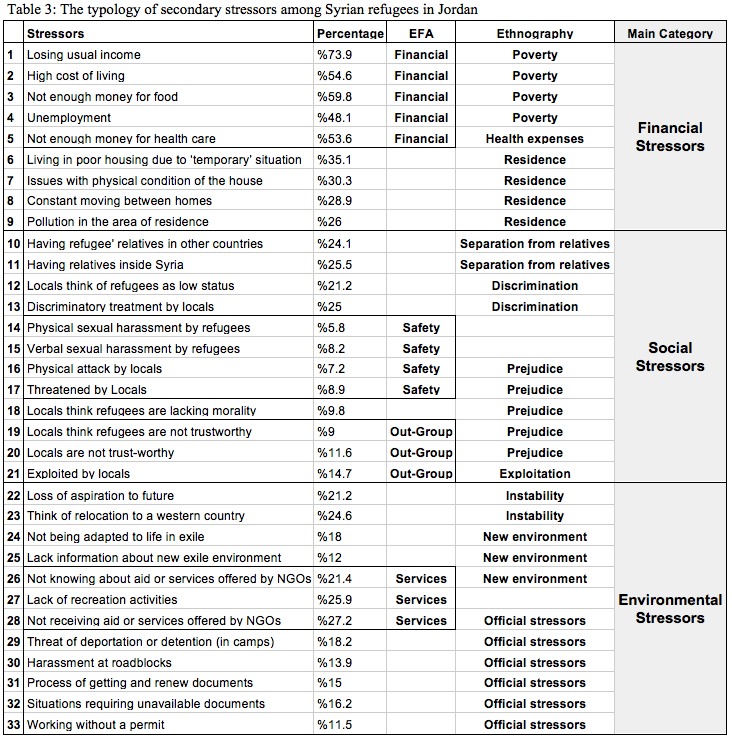

In the typology, we divided the stressors into main themes and then to smaller categories in an attempt to explore the relation between different stressors, but in reality, such division is not pure as overlaps are expected and the nature of a specific stressor can arguably fall under more than one possible category. Our main reason for creating the typology was to identify the main sources of secondary stressors. As we argued in the introduction, by definition secondary stressors arise from the exile environment and thus are environmental in nature, and socially mediated which emphasises the social nature of such stressors; and we acknowledge financial ability to buffer many stressors in situations like forced displacement. However, we focused on the main source making the stressor most salient in the refugee’s experience, which is either the structure which forces conditions collectively on refugees (e.g., documentation restrictions/environmental stressors), or social interactions with individuals that spark stress (e.g., exploitation), or the lack of financial ability that determines accessibility to services (e.g., advanced healthcare). Thus, we decided to consider residence, education, and health expenses stressors as financial stressors because these are the “stressors of the poor”. We considered worrying about relatives in Syria as a secondary stressor instead of treating it as a primary stressor, directly caused by war, because we believe that the main stress source in this case is the separation itself, not to the dangerous situation that the relatives are experiencing, which we think is also relevant. What gives us confidence that the mere separation from family is the main source of stress is that the participants were found to worry about relatives in Syria (25.4%) to a similar degree that they worry about relatives in other countries (24.1%).

**Financial stressors**

This type of stressor is the most important, as it was clearly found in both qualitative and quantitative analysis, and the top five stressors common among the participants were all financial (see Table 1). This came as no surprise due to the fact that most of the refugees lost their income and homes and depleted their savings during many years in exile, and due tothe high living expenses in Jordan compared to Syria or other neighbouring countries. Two specific financial stressors (poor housing condition and low quality of education) did not group well with the other stressors (maybe because these two result from them), although a considerable number of the survey participants (30% and 17.7%) reported suffering from these stressors.

**Environmental stressors**

This category included stressors that are circumstantial either in the structure of the exile environment (e.g., services and legal requirements) or relate to a general feeling created by this environment (e.g., instability and lack of familiarity). Most Syrian refugees - especially those who were displaced early in the crises - left their homes as a temporary solution and were not prepared (financially or mentally) for many years in exile with an ongoing civil war without an end in the horizon. This situation is complicated by the fact that Jordan is not a member of the 1951 Refugees Convention, which ensures basic services to refugees (e.g., housing, work, and education)^5^. The Syrian refugees could face these harsh conditions indefinitely as Jordan is very clear in its resistance to refugees being integrated (there are still Palestinian refugee camps in Jordan since 1967). Syrian refugees know that they cannot stay in Jordan or go back home because of war, and relocation to a Western country is not an option for the majority of the refugees because these countries only take a small percentage of refugees^1^ and additionally many refugees are not interested in living in a Western country which takes them far from their relatives and traditional culture.

**Social stressors**

This group of stressors directly arises from social relations, either with locals or other refugees, or the lack of social interaction in the case of separation from relatives. The stressors arising from social interactions in exile were found to be grouped into two types: stressors related to relations with locals and safety stressors.

In the interviews, most of the refugees said that they have good relations with Jordanian people and that they appreciate the efforts and services offered to them in Jordan. However, some refugees reported occasional issues in their relationships with locals (prejudice and exploitation) and more common issues in regard to systematic discrimination against them (e.g., work restrictions, lower quality education and multiple official document requirements). This analysis was supported by the quantitative analysis, which showed that discrimination against Syrian refugee appears to be more common (25% of the participants) compared to experiencing prejudice (less than 15%).

The social violence (safety) group of stressors (physical and verbal sexual assaults) is important, as it represents how the stressors of the exile environment can be traumatic, and at the same time does not have to be common or “daily”. This supports our argument for using the term “secondary stressors” to describe these stressors arising from the exile environment instead of “daily stressors” that is widely used in contrast to traumatic stressors.

## Limitations

Both studies reported here were conducted using a convenience sample of adult Syrian refugees in Jordan, and thus we should not assume it is representative of the general population of Syrian refugees in Jordan. Assumptions based on our findings should be examined among larger and more diverse samples of refugees in the Middle East, which will also help to refine the data collection and measurement tools used in both studies. By definition, secondary stressors arise from the exile environment, and thus we predict different secondary stressors among Syrian refugees in other countries, due to the difference in the environment (e.g., the language barrier to Syrian refugees in Turkey). Therefore, we need to extend our examination to include more hosting countries in the region as they have different situations (e.g., financial, security situations and demographics) that could shape the exile into an environment different than what we examined in Jordan.

We recommend more exploration among more refugees in more locations in order to produce more comprehensive and more reliable typology of secondary stressors among refugees of conflict in the Middle East and developing countries in general. Although we laid a foundation to build a scale for secondary stressors in the region, it still needs to be tested it in a wider sample and the items refined.

## Conclusions

As shown in the introduction, the relief literature does recognise the importance of stressors arising from exile, but it lacks an organised conceptual framework to classify these stressors or to measure them. The only effort that we are aware of is a typology of secondary stressors in disasters,^19^ which was not designed with armed conflicts in mind. We recommend using the term secondary stressors instead of daily stressors, as it includes stressors that are not daily in nature, in addition to traumatic stressors that arise from exile. Our data showed that refugees did occasionally report primary stressors caused by war such as the loss of relatives or property back home, but they clearly emphasised that the main challenges were secondary stressors. Some of the secondary stressors reported by the participant were quite serious and could be considered traumatic (e.g., physical and sexual attacks) which again suggests the flawed use of the term daily stressors, especially when used in contrast with traumatic experiences.

We found that Syrian refugees in Jordan suffer the most from financial stressors, due to the loss of income and high living expenses, and deficiency of the services available to them. Social stressors were found among a considerable number of refugees, where they varied from a group of stressors due to being targeted as a refugee by the locals/government (e.g., discrimination) to more traumatic stressors that came from both locals and other refugees (e.g., assault).

We hope that this typology will offer help to both interventions and future research to identify and classify secondary stressors among refugees of conflicts.

## DATA AVAILABILITY

The survey data are available on Figshare at DOI: 10.25377/sussex.6169316. The interview transcripts contain sensitive data and cannot be anonymized, therefore they are available upon request. These restrictions are imposed by the Sciences & Technology Cross-Schools Research Ethics Committee at University of Sussex. Interested researchers can request access to the data from the Sciences & Technology Cross-Schools Research Ethics Committee at University of Sussex at crecscitec@sussex.ac.uk.

## COMPETING INTERESTS STATEMENT

The authors have declared that no competing interests exist.

## CORRESPONDING AUTHOR

Khalifah Alfadhli who may be contacted at alfadhli@ksu.edu.sa.
